# Unrewarded Object Combinations in Captive Parrots

**DOI:** 10.12966/abc.11.05.2014

**Published:** 2014-11-01

**Authors:** Alice Marie Isabel Auersperg, Natalie Oswald, Markus Domanegg, Gyula Koppany Gajdon, Thomas Bugnyar

**Affiliations:** 1 University of Vienna; 2 University of Oxford; 3 Karl-Franzens-University; 4 University of Vienna and University of Veterinary Medicine; 5 University of Veterinary Medicine; 6 Tiergarten Schönbrunn; 7 MaxPlanck Institute

**Keywords:** Object play, Exploration, Object combinations, Avian cognition, Parrot

## Abstract

In primates, complex object combinations during play are often regarded as precursors of functional behavior. Here we investigate combinatory behaviors during unrewarded object manipulation in seven parrot species, including kea, African grey parrots and Goffin cockatoos, three species previously used as model species for technical problem solving. We further examine a habitually tool using species, the black palm cockatoo. Moreover, we incorporate three neotropical species, the yellow- and the black-billed Amazon and the burrowing parakeet. Paralleling previous studies on primates and corvids, free object-object combinations and complex object-substrate combinations such as inserting objects into tubes/holes or stacking rings onto poles prevailed in the species previously linked to advanced physical cognition and tool use. In addition, free object-object combinations were intrinsically structured in Goffin cockatoos and in kea.

Playful object manipulation outside a foraging context (Fagen, 1981) can reflect cognitive abilities contributing to a survival. Thus, the degree of structure and complexity within the manipulation can vary substantially across different species. In primates, object manipulation can roughly be subdivided into either simple object actions (primary manipulations) such as picking up an object using one appendage or complex object actions (secondary manipulations), during which the object is moved in relation to specific environmental features, including the manipulation of an object using several appendages at once, bringing an object into direct contact with another object or orienting an object in relation to a substrate (e.g., Hayashi & Matsuzawa, 2003; Hayashi, Takeshita, & Matsuzawa, 2006; Takeshita, 2001; Takeshita & Walraven, 1996; Torigoe, 1985). Within complex object actions, object-object as well as object-substrate combinations are largely considered critical precursors of functional behaviors such as flexible tool use or overall technical problem-solving skills in a number of primate species and have also been revealed to play a significant role in the development of tool use in human toddlers (e.g., Connolly & Dagleish, 1989; Fragaszy & Adams-Curtis, 1991; Fragaszy, Fedigan, & Visalberghi, 2004; Graham & Burghardt, 2010; Hayashi et al., 2006; Huffman, 1996; Rat-Fischer, O’Regan, & Fagard, 2012).

Previous studies suggest that amongst a large number of primates investigated, complex object-object and object-substrate combinations seem to be mainly restricted to capuchin monkeys and the great apes (Fragaszy et al., 2004; Hayashi et al., 2006; Torigoe, 1985). The latter have repeatedly exhibited advanced cognitive skills in the technical domain and are also habitually tool using species (Shumaker, Walkup, & Beck, 2012). In birds, both corvids and parrots have repeatedly shown performance levels equivalent to the great apes in various problem-solving tasks and some species have even innovated flexible tool use in experimental settings (e.g., Auersperg, Gajdon, & Huber, 2010; 2011; Auersperg, von Bayern, Gajdon, Huber, & Kacelnik, 2011; Auersperg, Szabo, von Bayern, & Kacelnik, 2012; Bird & Emery, 2009; Cheke, Bird, & Clayton, 2011; Gajdon, Aman, & Huber, 2011; Taylor, Hunt, Holzhaider, & Gray, 2007; von Bayern et al., 2009; Weir, Chapell, & Kacelnik, 2002). So far there have, however, been only few investigations into unrewarded object-object and object-substrate combinations in these birds.

Kenward et al. (2011) compared the development of object combinations in fledglings of the habitually tool using New Caledonian crow (*Corvus moneduloides*) and of common ravens (*Corvus corax*). They found that throughout their study period object combinations increased steadily in New Caledonian crows but peaked and declined in common ravens. This supports the assumption that combining objects with other objects or substrate properties during juvenile play may have promoted the evolution of habitual tool use in the New Caledonian crow. Furthermore, another experiment, the predecessor of the present study (Auersperg et al., in press), investigated unrewarded object combinations in three corvids, including the habitually tool using New Caledonian crow and three parrots. They compared two neotropical parrots, the Hahn’s macaw (*Diopsittaca nobilis*) and the black-headed parrot (*Pionites melanocephala*), which are currently used as model species in technical experiments and the Goffin cockatoo (later Goffin, *Cacatua goffini*), which has shown the capacity for several categories of tool use and manufacture in laboratory experiments (Auersperg et al., 2012; Auersperg, Seyerl, von Bayern, & Bugnyar, unpublished data). Similar to the New Caledonian crows, Goffins displayed several types of combinatory object actions such as combining up to three free objects and coordinated object-substrate combinations such as inserting objects into tubes and holes or stacking rings onto poles or sticks. The Goffins’ actions parallel previous observations in the habitually tool using primates and carry some structural elements of object play in human infants (Connolly & Dagleish, 1989; Langer, 1986; Torigoe, 1985).

Correspondingly, Pepperberg and Shive (2002) investigated unrewarded, free object-object combinations in the African grey parrot (later GP, *Psittacus erithacus*), which can acquire highly progressive categorization abilities in human language experiments (see Pepperberg & Shive, 2002 for a summary). GPs additionally showed advanced object knowledge in several technical tasks (Pepperberg & Kozak, 1986; Pepperberg, Willner, & Gravitz, 1997; Péron, Rat-Fischer, Lalot, Nagle, & Bovet, 2011; Schloegl, Schmidt, Boeckle, Weiß, & Kotrschal, 2012) and there have been observations reporting self-directed tool use in a non-foraging context (using an object to scratch the own body) in captive animals (Boswall, 1977; Janzen, Janzen, & Bond, 1976). The four-year-old subject tested by Pepperberg and Shive (2002) combined objects in distinctive ways, stacking them on top of one another, potting smaller objects into larger ones or placing two objects next to one another.

Another parrot, which combines objects in a non-foraging context, is the extremely generalist and neophilic kea (*Nestor notabilis*). In New Zealand’s national parks kea are frequently reported to engage in novel object manipulation, destroying parts of tourist cars and camping equipment (Diamond & Bond, 1999). In captivity, kea do not only seem to use objects to create effects, carrying them around and smashing them on different substrates, they also engage in unrewarded insertion of objects into tubes and other objects and combine free objects (Gajdon, Lichtnegger, & Huber, 2014). The kea’s playfulness, which, at least in captive animals, seems to be maintained throughout adulthood, may help them to cultivate a generalized toolkit of behaviors that they can flexibly employ when faced with unexpected foraging problems (Diamond & Bond, 1999). Similarly to the Goffin, the kea has also shown the capacity for using different tools in various laboratory setups but is not known to be a habitually tool using species (Auersperg et al., 2010; Auersperg, Gajdon et al., 2011; Auersperg, von Bayern et al., 2011; Gajdon et al., 2011; Gajdon et al., 2014)

In order to gain an enhanced synopsis over object combinations in parrots we use an experimental setup formerly applied to Goffins and two neotropical parrots (Auersperg et al., In Press) to a larger set of species. Our study targets differences within and between species in combinatory actions including (1) free object-object combinations (bringing two free objects into direct contact) (2) object-substrate combinations (placing free objects into operationally complex relationships with fixed substrate structures e.g., inserting objects into tubes or holes, stacking rings onto poles or tubes, recovering objects from poles, tubes or holes).

We concentrated our choice in part on species which have previously gained attention for advanced abilities in the technical domain, such as the GP, or have been associated with tool related behavior in experimental settings, such as the kea. Moreover, as we had access to Goffin data from Auersperg et al. (in press), we reprocessed it in the present study. We additionally wanted to include a habitually tool using parrot: Australia’s black palm cockatoos (BPCs, *Probosciger aterrimus*) are unique as they habitually use tools for a social purpose. They beat pieces of wood or hard nutshells with their feet against perches or hollow trees (Lantermann, 1999; Rowley, 1997). This behavior is predominantly exhibited by males during the breeding season in order to direct the females’ attention to potential breeding holes or as territorial display (Lantermann, 1999). BPCs are presently the only parrots that are regularly observed to use tools in the wild (although anecdotal evidence indicates that Hyacinth macaws, which use tools in captivity, may also do so in their natural habitat; Borsari & Ottoni, 2005). We further picked three further neotropical parrots; the two Jamaican Amazon species, the black-billed amazon (later BBA, *Amazona agilis*) and the yellow-billed Amazon (later YBA, *Amazona collaria*) which is closely related to the former but has a much wider distribution. The YBA can be found in a number of habitats, including cultivated areas (Collar, 1997; Wiley et al., 2004) while the BBA is restricted to moist forests (Collar, 1997). We also tested burrowing parakeets, inhabitants of Argentina’s vast grasslands (Forshaw, 2006).

As all parrots tested are large brained and social birds with relative slow maturation we expect subjects of all species to engage in various types of primary, simple play patterns (e.g., Diamond & Bond, 2003; Diamond, Eason, Reid, & Bond, 2006). Species with the capacity for tool use or which have previously shown advanced skills in the technical domain are expected to engage in complex manipulations such as object-object or object-substrate combinations and to show structural elements within the former.

## Method

### Subjects

We had the opportunity to test two BPCs. One (Kathy) was parent raised in 1986, while the other (Madame D.) was hand-raised in 2008. Both were housed together at the Vienna Zoo (Tiergarten Schönbrunn). We observed 14 kea, which were kept as a single group at the Haidlhof Research Station in Lower Austria. Twelve kea were adult (three hatched in 2004, seven in 2007 and two were older than 10), two were sub-adult (2008 and 2010 respectively). Five kea were parent-raised (four at the Konrad Lorenz Institute for Ethology in Vienna and one at the Vienna Zoo) and nine hand-raised (all at the Konrad Lorenz Institute for Ethology). Furthermore, ten GPs took part in this experiment. All available subjects were adult but not senior, with hatching dates from 1990 to 2004. GPs were kept as a single group at the Vienna Zoo, five were parent-raised and hatched at the zoo and two were hand-raised. The rearing history of three birds was unknown. We also tested two species of Jamaican Amazon parrots. All had hatched and were hand-raised at the Vienna Zoo in 2011 and had been kept there since in single species group enclosures. Fifteen YBAs and six BBAs took part in this study. Finally, three juvenile and nine adult burrowing parakeets were tested. All (but one adult which derived from private owners) were transferred from Budapest zoo to Vienna Zoo in 2004. All juveniles hatched in May 2012 at the Vienna Zoo. All burrowing parakeets were parent-reared and were kept in a social group.

We additionally included Goffin data from a previous experiment using essentially a matching methodology (Auersperg et al., In Press). This was done to increase the sample of species with a captive capacity for flexible tool use. At the time, ten juvenile (eight hatched 2010 and two in 2011) and four adult Goffin cockatoos (two hatched in 2007 and two in 2008), were tested. All Goffins were kept as a group at the ‘Goffin Lab’ of the Department of Cognitive Biology (Lower Austria).

All parrots (except for kea which were fed twice a day) were kept at ad libitum diet (fresh and dried fruits and vegetables, minerals, a selection of cooked and uncooked seeds, various protein sources and fresh drinking water) in enriched housing conditions. All aviaries included enriched outdoor and heated indoor areas (except for kea which thrive in colder climates and were kept in a large outdoor-only aviary with shelters). For more information on all parrots (aviary sizes, time frame of testing, observer, etc.) see Table 1.

### Experimental History

The Goffin cockatoos had previously participated in a means-means-end task featuring an artificial fruit apparatus (Auersperg, Kacelnik et al., 2013) as well as in an experiment on Piagetian object permanence (Auersperg, Szabo et al., 2013) but had not been tested in the context of object play, or previously presented with the objects used in this experiment. Most kea except for two subjects: an adult parent-reared female, Elvira, and a juvenile parent-reared male, Paul, had participated in various cognitive experiments including inserting as means task, which required the insertion of objects into tubes (e.g., Auersperg et al., 2010; Gajdon et al., 2011). The inexperienced kea Elvira was the only kea which did not interact with the objects, she was however low in rank and over ten years old at the time of testing. The kea Paul and Elvira may have witnessed general unrewarded object play of their group mates. None of the remaining subjects had previously actively participated in cognitive experiments, or were previously presented with the objects used in this experiment.

### Ethics Statement

All animals are permanently kept in well-established groups at the respective research institutions as well as the Vienna Zoo and are housed firmly in accordance with Austrian Law. Testing was discussed and approved by the institutional ethics committee in accordance with Good Scientific Practice guidelines and national legislations As our experiments are strictly non-invasive and based purely on behavioral observations, they cannot be classified as animal experiments in accordance with the Austrian, Animal experiments Act (§ 2. Federal Law Gazette No. 501/1989).

### Apparatus

**Figure 1:**
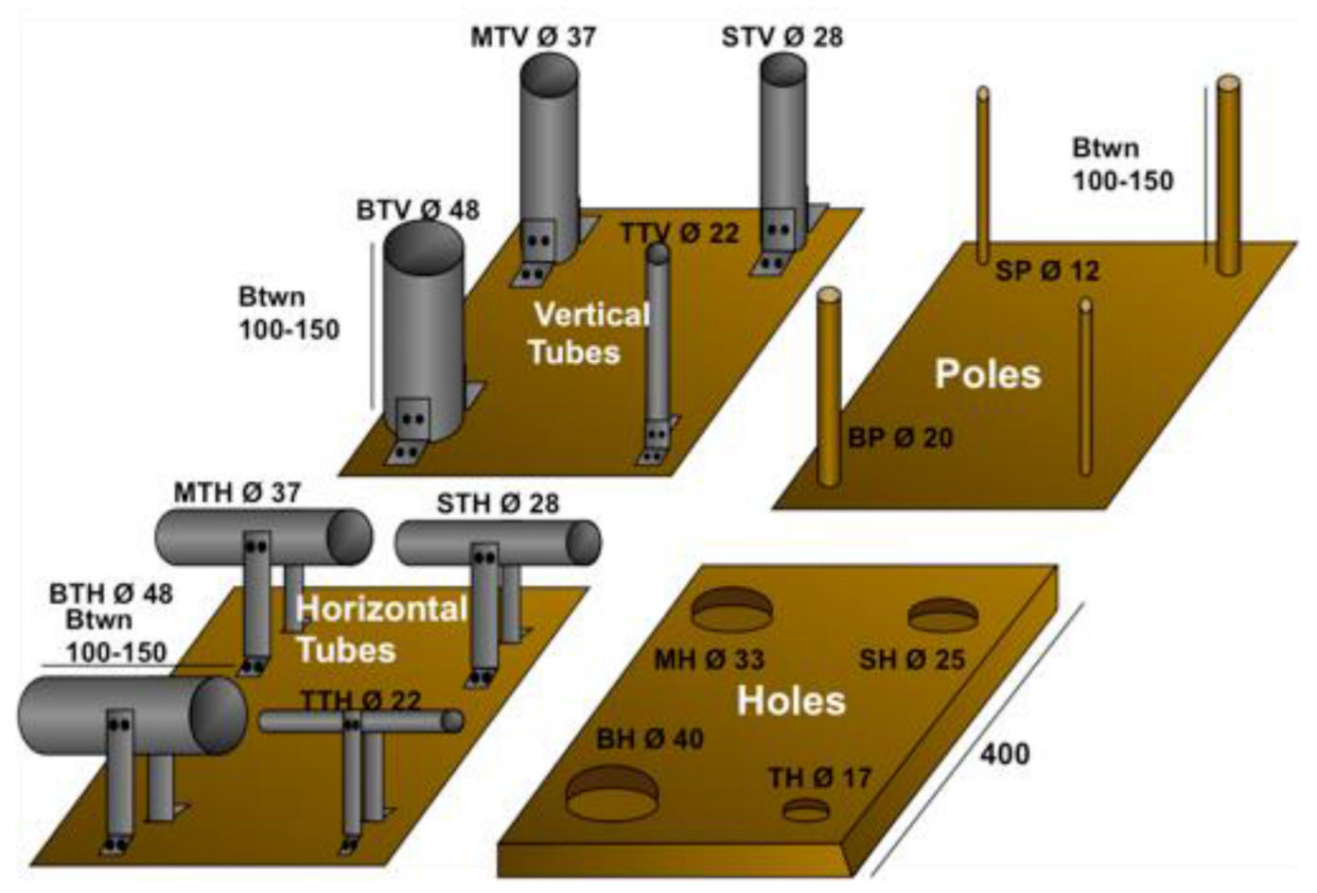
Activity Plates (dimensions in mm): tubes and poles (length 80, 100 or 150 mm; 100 for Goffins, 150 for kea, BPC); vertical tubes: big tube vertical (BTV), medium tube vertical (MTV), small tube vertical (STV), tiny tube vertical (TTV); horizontal tubes (BTH-TTH); poles (BP & SP); holes (BH-TH). BTH, BTV and BH fit all objects except big rings, MTH, MTV and MH fit all objects except for the big rings cubes and balls, STH, STV and SH fit all objects except for big and medium rings, cubes and balls. TTV, TTH and TH fit sticks from size S2-S5. As in Auersperg et al. (in press), in agreement with the authors of the previous study.

Four ‘activity plates’ with holes and tubes of different diameters were placed on the floors of the aviaries. The activity plates comprised of: (1) four horizontal tubes, (2) four vertical tubes, (3) a 30-mm thick plate with four holes drilled into the wooden base and (4) four dowel poles of two different diameters (see Figure 1 for dimensions).

**Figure 2:**
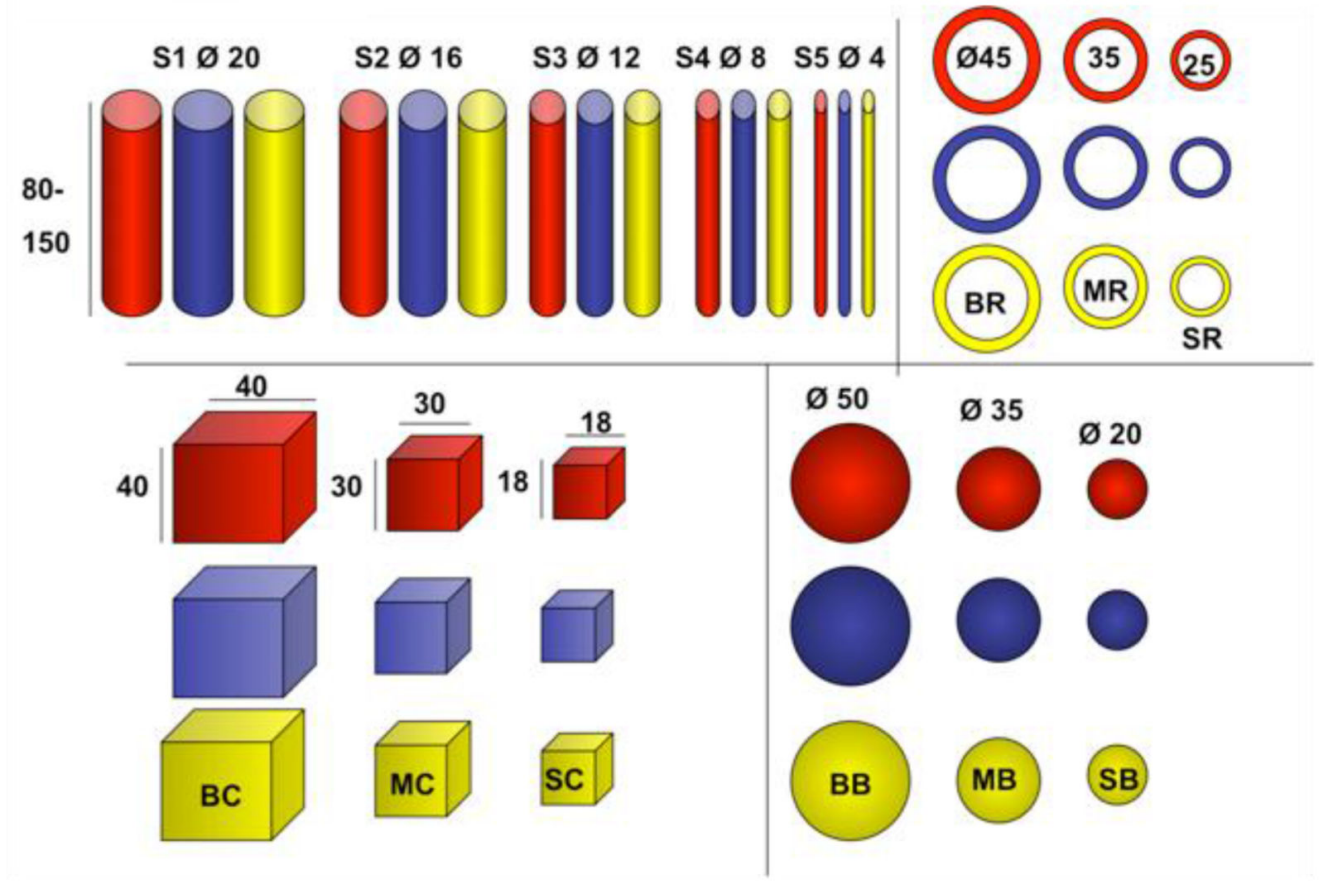
Wooden objects (dimensions in mm); Cubes: Big Cube (BC), Medium Cube (MC), Small Cube (SC); Balls (BB-SB); Rings (BR-SR). Sticks (S1-S5) were 80, 100 or 150 mm long (100 for Goffins, 150 for kea, BPC). Goffins and kea initially had a smaller sized ball (10 mm diameter), which was not licensed to be offered to zoo parrots. As in Auersperg et al. (in press), in agreement with the authors of the previous study.

At the same time, we presented subjects with three sets of the same wooden objects each painted in yellow, red and blue using childproof, non-toxic, water-based acrylic paint. Four of the five shapes (cubes, balls, and rings) came in three sizes, one (sticks) in five (see Figure 2 or Appendix B for dimensions).

### Experimental Setup and Procedure

The activity plates were arranged on the aviary floor in a rectangular organization at approximately 40-50-cm distance from one another (as in Figure 2). The position of each activity plate varied randomly across the sessions. During a habituation phase, the plates were placed on the aviary floor (without the corresponding objects) until they had been haptically manipulated by the birds (due to different levels of neophobia, this phase varied from a few seconds for kea to over several days for burrowing parakeets). Prior to testing the objects were spread randomly around the activity plates at 50-cm distance to the arrangement. To avoid constraining play behavior and causing stress, we tested all animals in their normal housing conditions in a group context. We conducted a total of 15 observations for each species with each session lasting for 30 min. After each observation, the objects were removed and presented again in the next session. To maintain motivation, we tested at two to three day intervals. The ethogram used contained solely object-related behaviors (see Appendix A) and was consistent across species. Object actions were recorded on a digital voice recorder and on video, starting from the moment the first object was touched by any subject within the group. If no subject touched any of the objects for 30 min, the objects were removed and the session was repeated in the next testing slot. If this happened for ten consecutive testing slots, we quit the experiment. Part of the data (five sessions of each, GPs, YBAs and BPCs) was double rated by both observers (NO and MD). Inter-rater reliability was adequate (Interclass correlation coefficient 0.87).

### Analysis

Since burrowing parakeets did not interact with the objects and only two BPCs were available to us, we did not include the respective data into the statistical analysis. Analysis focused on the object actions summarized in Table 2. We used the mean frequencies or mean percentages of the respective actions with objects as recorded across subjects for each species for non-parametric analysis (data failed to meet the criteria required for parametric analysis).

We conducted General Linear Mixed Models (GLMM) over the following object actions: free object-object combinations, object-substrate combinations (combinatory actions with objects at the activity plates) and activity plate preferences. We continuously used ‘subjects’ as random factor and ‘species’ and ‘sex’ as fixed factors. For the GLMM ‘activity plate preferences’ we used ‘activity plate’ (horizontal tubes/vertical tubes/holes/poles) and for the GLMM ‘object-substrate combinations’, ‘type of object-substrate combination’ (inserting/ring-stacking/recovering) as a third fixed factor. If a species performed a behavior less than five times in total we did not include it into the respective model.

Significant outcomes were analyzed post-hoc using non-parametric paired tests (Wilcoxon tests and Mann-Whitney U tests). The outcomes were corrected depending on the number of comparisons using the Bonferroni-Holms method.

## Results

All species except for burrowing parakeets interacted repeatedly and variably with the experimental objects within the time frame of this study. In GPs only the two hand-raised subjects (who are also an affiliated pair) repeatedly interacted with the objects. The occurrence of free object-object and object-substrate combinations varied within and between the remaining species (see below). BBAs frequently manipulated the experimental objects but never showed any of the given types of combinatory actions throughout the experiment.

### Color/Shape Preferences

In order to control for baseline preferences we looked at category choice during general object manipulations (including manipulations in which the objects were not combined). The respective analysis is detailed in the Appendix (Section C). In brief, BPCs preferred red objects. Goffins interacted more with yellow objects than with red, kea picked yellow over red and blue objects. Goffins further chose sticks more than cubes, kea balls more than rings, YBAs sticks, balls and cubes more than rings and GPs picked sticks over all other objects.

### Free Object-Object Combinations

**Figure 3:**
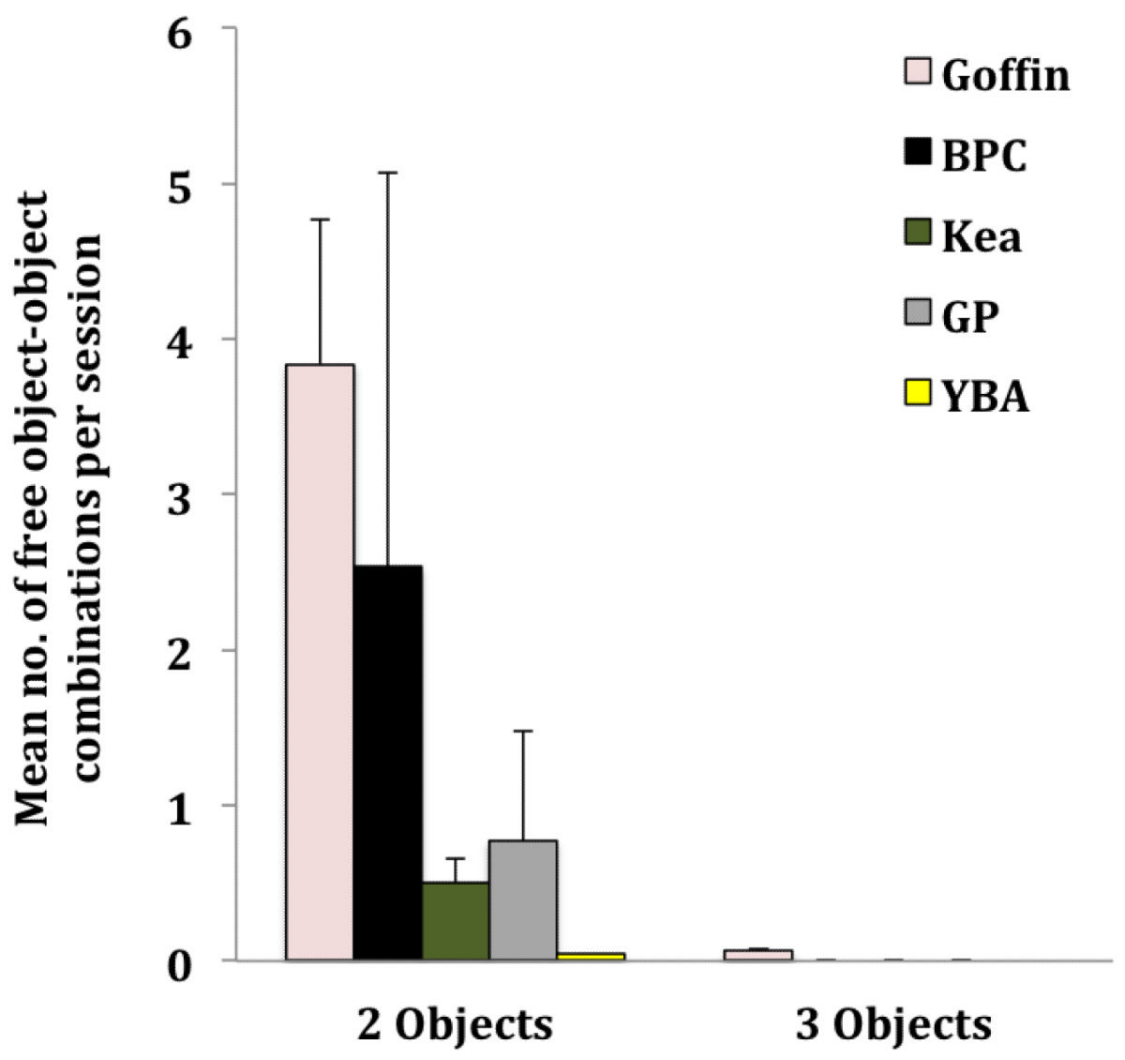
Mean number (± SE) of free object-object combinations of two objects (left) and of three objects (right) per session. The colors of the bar charts indicate different species: rose = Goffin (*N* = 14), black = BPC (*N* = 2), olive = kea (*N* = 14), grey = GP (*N* = 10), yellow = YBA (*N* = 15).

We only included data from Goffins, kea, GPs and YBAs into the GLMMs for free object-object combinations. The younger BPC Madame D. did combine two free objects as often as 76 times throughout the experiment but this was limited to one subject (see Figure 3).

We found differences in the mean frequency of object-object combinations between the four remaining species (Goffins, kea, GPs and YBAs; GLMM, *F* = 9.462, *df* = 3, 45, *p* = 0.0001) but neither between the two sexes nor an effect of an interaction species*sex. Post-hoc tests revealed that Goffins combined two objects more often than kea (Mann-Whitney U test, *Z* = 3.472, *p* < 0.0001), GPs (Mann-Whitney U test, *Z* = 3.28, *p* = 0.001) and YBAs (Mann-Whitney U test, *Z* = 4.619, *p* < 0.0001). Kea combined free objects more often than YBAs (Mann-Whitney U test, *Z* = 3.4, *p* = 0.001). No other significant species differences were uncovered (Mann-Whitney U tests, *p* values were above initial value of respective Bonferroni threshold = 0.008). Note that there were only two GPs which interacted with the objects more than once. One combined two objects 107 times, the other nine times. If we chose to disregard the remaining GPs, their average would be higher than kea (7.5 ± 2.26 SE) and similar to Goffins (57.57 ± 13.9 SE) and BPCs. Only Goffin cockatoos combined three free objects (see Figure 3).

### Object-Substrate Combinations

#### Types of object-substrate combinations

**Figure 4:**
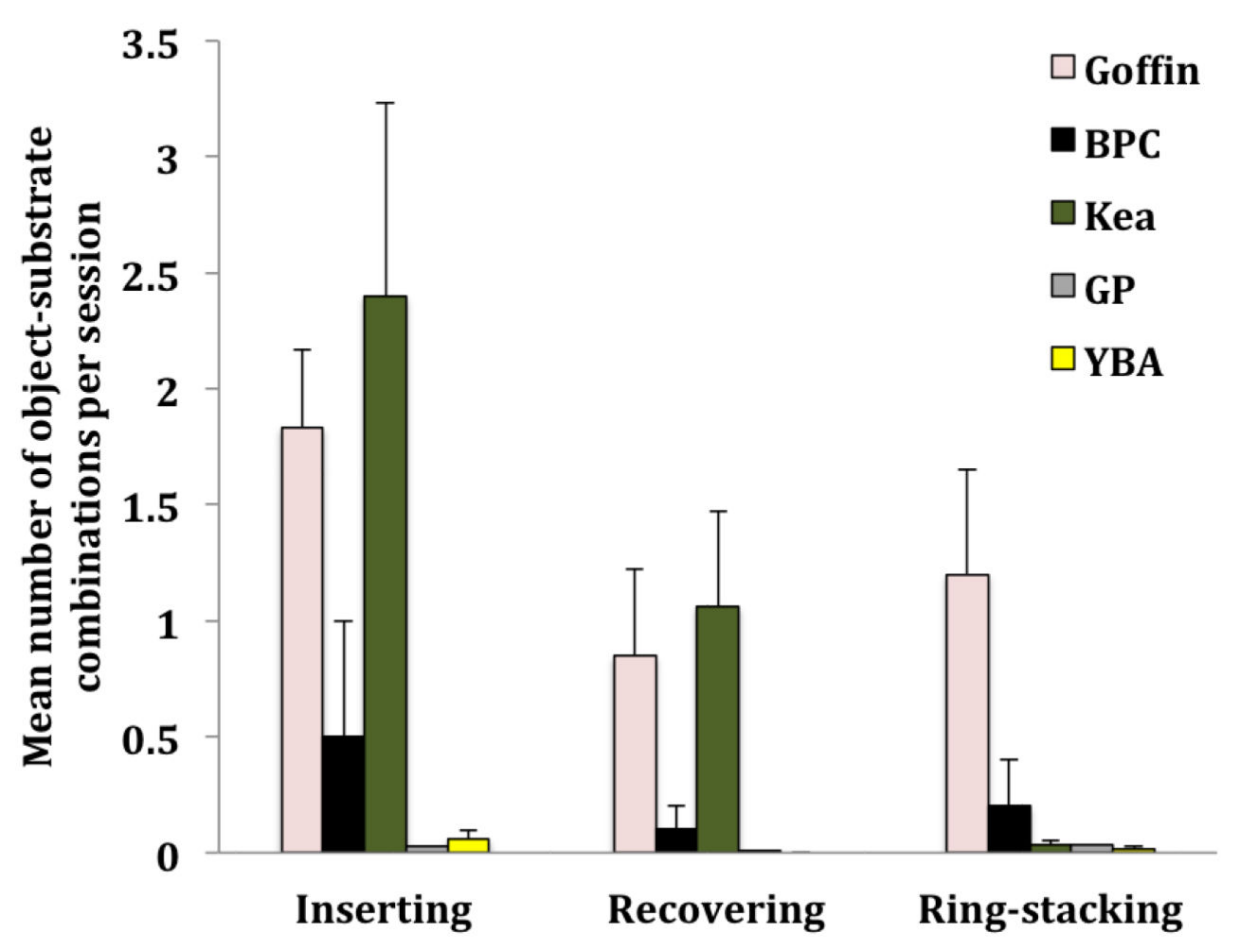
Mean number (± SE) of inserting, recovering and ring-stacking actions per session. The colors of the bar charts indicate different species (Note that objects can be recovered after both, ring-stacking and inserting instances): rose = Goffin (*N* = 14), black = BPC (*N* = 2), olive = kea (*N* = 14), grey = GP (*N* = 10), yellow = YBA (*N* = 15).

The BPC Madame D. inserted objects into the tubes and holes 14 times; she stacked rings onto tubes and poles six times and recovered objects from the tubes, poles and holes four times. The older subject (Kathy) never combined objects with the activity plates (see Figure 4). The two GPs which did interact with the objects each inserted objects into rings up to two times and one subject stacked rings onto free sticks five times, but as these actions remained at low levels and as they failed to interact with the activity plates they are not included into the following analysis.

The GLMM detected differences between the remaining species (Goffins, kea and YBAs) that combined objects with the activity plates in the described manner (GLMM, *F* = 5.204, *df* = 2, 117, *p* = 0.007). We also found differences in the frequency of the three different types of object/plate combinations investigated (*F* = 11.646, *df* = 2, 117, *p* < 0.0001) and an effect of an interaction species*type of object/plate combination (*F* = 5.502, *df* = 4, 117, *p* < 0.0001). We found no effect for sex and no effect of an interaction sex*species.

In Post-hoc tests we found that kea and Goffins inserted more objects than YBAs (Mann-Whitney U tests, *Z*_kea_ = 3.611, *p* = 0.001; *Z*_Goffin_ = 4.423, *p* < 0.0001). Only Goffins and kea recovered objects from the tubes/poles. Goffins, kea, and YBAs stacked rings on poles, tubes or other toys. Goffins did this more than any of the other two species (Mann-Whitney U tests, *Z*_Kea_ = 4.06, *p* < 0.0001; *Z*_YBA_ = 4.444, *p* < 0.0001; see Figure 4). There were no differences between the remaining species (Mann-Whitney U tests, p value above initial value of the respective Bonferroni threshold = 0.008).

Looking at differences between the three types of object-plate combinations, Goffins inserted more objects into the tubes and holes than they recovered (note that objects can be recovered after both, inserting and ring-stacking incidences; Wilcoxon test *Z* = 2.413, *p* = 0.016) and kea inserted more than they stacked (Wilcoxon test *Z* = 2.934, *p* = 0.003) or recovered (Wilcoxon test *Z* = 2.803, *p* = 0.005). No other significant differences between the three types of activity plate combination could be found (Wilcoxon tests, *p* values above the initial value of the respective Bonferroni threshold = 0.0167). Notably, the juvenile kea Paul which lacked pre-experience from inserting objects into tubes inserted objects into tubes and holes just as much as the other kea (Paul: 53 insertions, kea with pre-experience on average: 34.7 ± 13.7 SE insertions).

#### Activity plate preferences during object-substrate combinations

**Figure 5:**
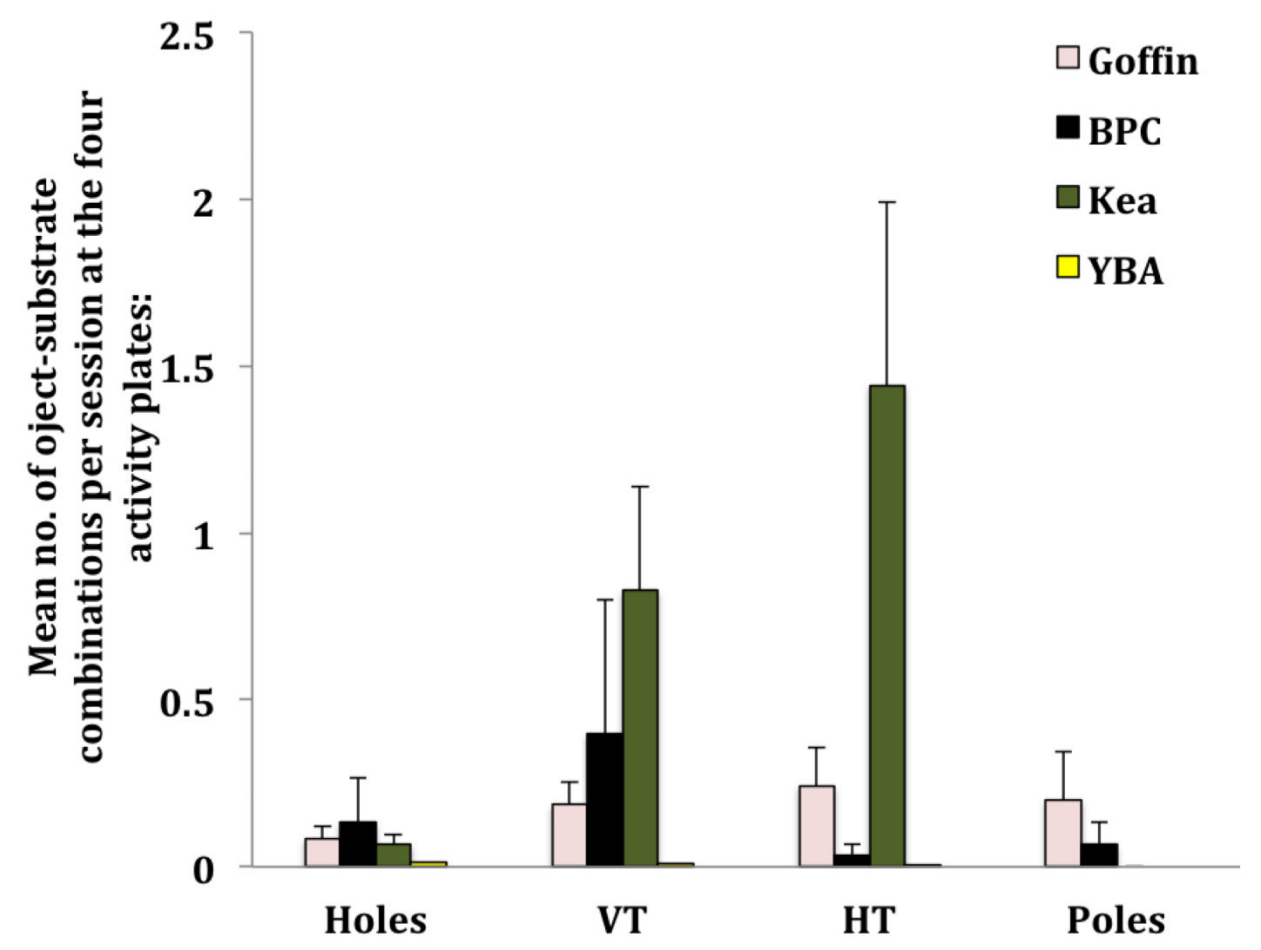
Mean number (± SE) of complex combinations per session at the four activity plates: Holes, VT = Vertical Tubes, HT = Horizontal Tubes, Poles. The colors of the bar charts indicate different species: rose = Goffin (*N* = 14), black = BPC (*N* = 2), olive = kea (*N* = 14), yellow = YBA (*N* = 15).

Of the BPCs, only Madame D. performed the previous combinations four times at the holes, 12 times at the vertical tubes, once at the horizontal tubes and twice at the poles (see Figure 5). Except for BPCs, only Goffins, kea and YBAs inserted/stacked/recovered objects from the activity plates. The respective GLMM detected differences in the choice of plate (GLMM, *F* = 6.844, *df* = 3, 157, *p* < 0.0001) between the species (*F* = 5.446, *df* = 2, 157, *p* = 0.005) and an effect of an interaction species* choice of plate (*F* = 5.634, *df* = 6, 157, *p* < 0.0001). We found no effect for ‘sex.’ Post-hoc we found that within species, kea preferred vertical tubes and horizontal tubes over holes (Wilcoxon test, *Z*_vertical tubes_ = 2.701, *p* = 0.007; *Z*_horizontal tubes_ = 2.803, *p* = 0.005) and horizontal tubes over poles (Wilcoxon test, *Z* = 2.936, *p* = 0.003). They did not stack rings on the poles. No significant activity plate preferences could be found for Goffins and YBAs (Wilcoxon test, *p* values above the respective Bonferroni threshold = 0.008).

Note that the inexperienced juvenile kea Paul inserted objects into the vertical tubes (Paul: 20 times, kea with pre-experience on average: 11.84 +/ 5.1 SE) and into the horizontal tubes (Paul: 23 times, kea with pre-experience on average: 21.5 +/ 9.068 SE) just as much as the kea who had rewarded pre-experience with tubes (Auersperg et al., 2010; Gajdon et al., 2011, 2014).

## Discussion

Several subjects within all species tested, except for burrowing parakeets, interacted with the objects within the given time frame. The latter, alongside the GPs, also took the longest to approach the activity plates during the habituation phase. At this stage, however, we do not have a plausible explanation for the parakeets’ apparent lack of interest in haptically exploring the given set of objects. New Zealand parakeets similarly failed to show aspects of combinatory object play (Funk, 1996). In GPs, only the two hand-raised birds manipulated the objects. In this case, rearing history may have influenced the level of novel object apprehension in this highly neophobic species. It has, for example, been shown that hand-reared juvenile orange winged amazons and New Zealand parakeets approach novel objects faster than parent-reared birds (Fox & Millam, 2004; Funk, 2002; Funk & Matteson, 2004). Nevertheless, it is curious that the remaining subjects did not even leave their perches throughout most of the entire experiment (15 sessions of 30 minutes).

All species that handled the objects, except for BBAs, included some object actions which could be categorized secondary/complex combinatory manipulations such as free object-object combinations or object-substrate combinations (Hayashi et al., 2006; Torigoe, 1985). We detected substantial discrepancies in the frequency, type and structural complexity of object actions within and between the remaining species.

### Color/Shape Preferences

Both BPCs seemed to prefer red objects. Interestingly, the otherwise all black BPC has unique red cheek patches that can change in color when the animal is excited (Forshaw, 2006). We also found a baseline preference for yellow in Goffins and kea. The otherwise all-white Goffins and the kea have yellow stripes underneath their wings, a body region commonly used during social displays; juvenile kea have yellow eye rings. Johnston (1999) previously found a yellow (and red) preference in wild kea, but not in captive ones. It is possible that such specific colors may carry significant information to the corresponding animals. Closer investigation would however be indispensable to confirm this. Four of the kea participating in this study previously participated in an experiment in which yellow tools were used (Auersperg, Gajdon et al., 2011). Previous rewarded experience with ball-shaped objects may also explain the respective preference in kea (Gajdon et al., 2014).

### Free Object-Object Combinations

Combinations of two free objects were not documented in BBAs but remained (at low frequencies) in YBAs. Wild BBAs are restricted to just one habitat type, while the closely related YBAs live a more general lifestyle, leaving the forests to expand to agricultural edge habitats (Wiley et al., 2004) and cultivated fruit crops (Collar, 1997), an ecological complexity which is arguably more cognitively demanding. Paralleling previous findings in primates and corvids (Auersperg et al., in press; Hayashi et al., 2006; Kenward et al., 2011; Torigoe, 1985), parrots previously associated with tool use and/or advanced abilities in the technical domain (BPCs, Goffins, GPs and kea) combined two free objects at relatively high rates and one of the latter (Goffins) even combined three items. Goffins and the habitually tool using BPCs (predominantly the sub-adult) seemed to combine free objects most frequently.

GPs and kea also combined two objects but at lower rates. However, only the hand-raised GPs interacted with the objects at all; if we considered only the two hand-raised GPs they would obtain similar results as BPCs and Goffins (see Figure 3), further supporting previous findings by Pepperberg & Shive (2002).

### Combinatory Actions with Objects at the Activity Plates

Inserting behaviors at the tubes and holes of the activity plates were largely limited to the BPCs, kea and Goffins (YBAs also inserted objects into tubes and holes but at very low levels). While BPCs are habitual tool users, both kea and Goffins are able to innovate several modes of flexible tool use. In both kea and Goffins all of the tool categories so far observed required inserting behaviors (Auersperg et al., 2010; Auersperg, Gajodon et al., 2011; Auersperg, von Bayern et al., 2011; Auersperg et al., 2012), indicating that inserting objects in a functional context could be anticipated through analogous patterns during object play (Gajdon et al., 2014). Although GPs have been reported to be capable of using tools, observations were so far limited to self-scratching and to one example of using a mirror as a tool to locate hidden objects (Boswall, 1977; Janzen et al., 1976; Pepperberg, Garcia, Jackson, & Marconu, 1995). Most available kea had rewarded pre-experience with inserting objects into vertically slanted tubes before this experiment (Gajdon et al., 2011; 2014). However, previous research on play in inexperienced kea indicated that inserting behaviors are also strongly expressed in a playful, unrewarded environment (Gajdon et al., 2014). Furthermore, the sub-adult kea Paul who lacked the experimental history of his aviary mates even inserted objects into the tubes and holes at a slightly higher rate. Kea, Goffins, the BPC Madame D. and YBAs stacked rings onto poles, tubes, or other toys; Goffins notably more often than the other species. Ring-stacking does not seem to be enacted by corvids (Auersperg et al., In Press). Parrots use their zygodactylous feet (Demery, Chapell, & Martin, 2011; Forshaw, 2006) during object manipulation, which may facilitate the sophisticated motor act of fitting a frame over another object. Probably as a consequence of their frequent inserting activity (and ring-stacking activity in Goffins) solely kea, Goffins and BPC Madame D. recovered objects from the tubes/poles.

### Activity Plate Preferences

Kea were the only birds which expressed a clear preference for horizontal tubes. This, again, may be a consequence of a rewarded pre-experience of inserting objects into horizontal tubes (Auersperg et al., 2010; Auersperg, Gajdon et al., 2011). However, since again, the naïve subject Paul performed in the same manner, there may be an alternative explanation: Qualitatively, the kea often rattled the horizontal tubes until they moved up and down on their fixation site. This may have generated a reinforcing effect (Gajdon et al., 2014, O’Hara, Gajdon, & Huber, 2012).

In conclusion, our observations strongly indicate a link between complex object combinations such as free object-object combinations and/or specific object-substrate manipulations and physical cognition in parrots: combinatory behaviors prevailed mostly in species associated with high-level problem solving skills and tool use. Two current model species for technical intelligence, the kea and the Goffin cockatoo, as well as the habitually tool using BPCs, displayed object-object and object-substrate combinations at high frequencies. GPs and only the Amazon species with the more variable habitat tested (YBA) also combined objects, in agreement with previous findings showing that avian innovation rates positively correlates with invasion success (Sol, Timmermans, & Leftbvre, 2002) In GPs this was limited to free object-object combinations while both free object-object as well as object-substrate combinations remained at relatively low levels in YBAs. Another, closely related Amazon with a more restricted ecology failed to combine objects in the course of our experiment and South American burrowing parakeets failed to even interact with the objects presented. Our results parallel previous findings on primates (Hayashi & Matsuzawa, 2003; Hayashi et al., 2006; Takeshita & Walraven, 1996; Takeshita, 2001; Torigoe, 1985) and corvids (Auersperg et al., In Press; Kenward et al., 2011), further implying that the respective cognitive substrates in large brained birds and primates have most likely evolved convergently.

## Figures and Tables

**Table 1:** Subject Details

Superfamily	Species	Sex	Age Class	Rearing	Housing	Where	Observer	Aviary Size	Time Frame
***Cacatuoidea***	**Goffin cockatoo** *(Cacatua goffini)*	7M, 7F	4 Adult, 10 Subadult-Juvenile	Hand	Group	Goffin Lab, Austria	AA	ID: 45m^2^, 2-6m high OD:150m^2^, 2-5m high	July-August 2011

	**Black palm cockatoo** *(Probosciger aterrimus)*	2F	1 Adult 1 Subadult	1 Hand 1 Parent	Pair	Tiergarten Schönbrunn Vienna	MD	ID: 22m^2^, 4m high OD: 12m^2^, 3m high	May-July 2013

***Strigopoidea***	**Kea** *(Nestor notabilis)*	8M, 6F	12 Adult, 2 Subadult	9 Hand 5 Parent	Group	Haidlhof Research St., Austria	NO	OD: 400 m^2^, 4m high	October-November 2012

***Psittacoidea***	**African grey parrot** *(Psittacus erithacus)*	6M, 4F	Adult	2 Hand 5 Parent 3 Unknown	Group	Tiergarten Schönbrunn Vienna	NO	ID: 22m^2^, 4m high OD: 24m^2^, 3m high	November-December 2012

	**Yellow-billed amazon** *(Amazona collaria)*	6M, 9F	Subadult	Hand	Group	Tiergarten Schönbrunn Vienna	MD	ID: 22m^2^, 3m high OD: 20m^2^, 3m high	February-March 2013

	**Black-billed amazon** *(Amazona agilis)*	2M, 4F	Subadult	Hand	Group	Tiergarten Schönbrunn Vienna	MD	ID: 11m^2^, 3m high OD: 10m^2^, 3m high	September-November 2013

	**Burrowing parakeet** *(Cyanoliseus patagonus)*	4M, 7F	9 Adult 3 Juvenile	Parent	Group	Tiergarten Schönbrunn Vienna	NO	ID: 9m^2^, 3m high OD: 78m^2^, 3m high	November-December 2012

Note. (Super-)family, species, sex (M=Male, F=Female), age class (juvenile/subadult/adult), rearing history (hand-reared or parent-reared), housing (group/pair), keeping location, observer (AA, NO, MD), aviary size (OD=outdoors, ID=indoors) and time frame in which data were collected.

**Table 2:** Subjects’ Object Actions that were used for Analysis

Object Actions	Factors
*Object Color Preferences*	Blue
	Yellow
	Red

*Object Shape Preferences*	Stick
	Ball
	Cube
	Ring

*Free Object-Object Combinations*	Combinations of two objects
	Combinations of three objects

*Object-Substrate Combinations*	Insertions (inserting, dipping, probing)
	Ring-stacking
	Recovering (from caches or insertions)

*Activity Plate Preferences*	Insertions into holes
	Insertions into vertical tubes
	Insertions into horizontal tubes
	Ring stacking onto poles

Note. Factors were compared within groups of actions for individual analysis and individually between species for interspecific comparisons.

**Appendix Figure S1:**
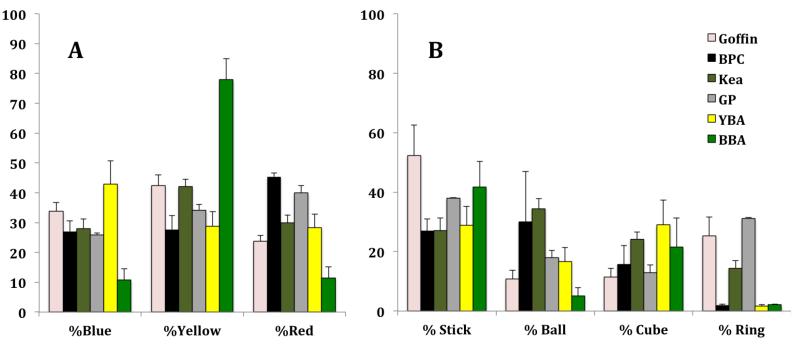
Mean percentage of object actions in which an object of a particular color (A: blue, yellow, red) or shape (B: stick, ball, cube, ring) was chosen. Goffin (N = 14), BPC (N = 2), kea (N = 14), GP (N = 10), YBA (N = 15), BBA (N = 6)

## Appendix A Object Manipulation Ethogram

### OBJECT-RELATED BEHAVIORS*

#### Free object-object combinations

■ *Balancing* (*ba-cb*): The bird is lying on its back and is balancing a combination of two or more objects with its feet.
■ *Carry* (*ca-cb*)*:* Holding two or more objects in the bill while the bird is moving along ground, or holding an object in feet while flying.
■ *Combining* (*cb*)*:* Placing one object into physical contact with another. Can be subcategorized as above and below. Ambiguous object-object combinations are coded as ‘cb.’
■ *Grab with foot* (*gf-cb*): Two or more objects are grabbed with the foot and simultaneously held up to the bill.
■ *Put on top* (*pot*): Placing one (or more) object on top of another object.
■ *Pick up* (*pi-cb*)*:* Two objects are seized together with the bill and lifted. Objects grabbed with the foot are coded as **‘**gf.’

#### Object-substrate combinations

■ *Caching* (*ch*)*:* Making an object disappear out of immediate sight. Includes putting objects underneath, between, behind and inside any aviary features except for the tubes, holes and poles on the activity plates or the aviary wire grid. NOT to be confused with ‘inserting’.
■ *Inserting*
  - Dipping (dp): Inserting or retrieving an object from an open cavity without releasing it (not to be confused with probing).
  - Inserting (in): An object is inserted into an opening or a hole in the activity plates or through the aviary wire cage walls (not to be confused with caching).
  - Probing (pr): inserting an object repeatedly into a cavity without letting it go.
■ *Ring-stacking* (*rst*): Placing a ring on top of another object e.g., placing a ring over a stick or over a pole (Objects inserted into rings lying on the floor are however coded as ‘inserting’).
■ *Recovering* (*rc*)*:* Recovering an object previously inserted or stacked upon the affordances of the activity plates (within a time frame of 20 s).

*Object actions involving only one item were recorded but not involved into the Analysis/ Results as presented in the manuscript.

## Appendix B Apparatus Dimensions

### Object abbreviations

Big Cube: bc

Medium Cube: mc

Small Cube: sc

Big Ring: br

Medium Ring: mr

Small Ring: sr

Big ball: bb

Medium ball: mb

Small Ball: sb

Stick 1 (thickest): (s1m*, s2e*)

Stick 2: (s2m, s2e)

Stick 3: (s3m, s3e)

Stick 4: (s4m, s4e)

Stick 5 (thinnest): (s5m, s5e)

*denotes stick grabbed at middle (m) or end (e)

All objects come in 3 colors: blue (b), red (r), yellow (y)

### Playground object abbreviations

Big Tube horizontal: bth

Medium Tube horizontal: mth

Small Tube horizontal: sth

Tiny Tube horizontal: tth

Big Tube vertical: btv

Medium Tube vertical: mtv

Small Tube vertical: stv

Tiny Tube vertical: ttv

Big Pole: bp

Small Pole: sp

Big Hole: bh

Medium Hole: mh

Small Hole: sh

Tiny Hole: th

### Dimensions wooden plates for tubes, poles and holes in mm

400×400, (300×300 for burrowing parakeets and grey parrots)

### Length tubes, sticks, poles in mm

80-150

### Inner diameter tubes in mm

TT 22

ST 28

MT 37

BT 48

### Diameter poles in mm

BP 20

SP 12

### Inner diameter holes in mm

TH 17

SH 25

MH 33

BH 40

### Inner diameter rings in mm

BR 45 (Thickness 10)

MR 32 (Thickness 10)

SR 20 (Thickness 0.7)

### Diameter sticks in mm

S1 20

S2 16

S3 12

S4 8

S5 4

### Diameter balls in mm

BB 50

MB 35

Sb 20

### Sidelength cubes in mm

BC 40

MC 30

SC 18

## Appendix C Object Color and Shape Preferences

To determine possible shape and color preferences for objects we noted (if clearly visible) the color/shape of each object subjects interacted with (this was not restricted to combinatory actions but included all object actions). For each subject we calculated the mean percentage of objects being of one of the three colors or one of the four shapes.

The BPCs slightly preferred red objects. 45.64% (Madame D.) and 44.14% (Kathy) of the objects they interacted with were red (see Figure S1). The two GPs that did interact with the objects did not seem to show preferences for a particular color. For the remaining species (Goffins, kea, YBAs and BBAs) the GLMM for color preferences indicated differences in the number of times the different colors were picked (GLMM, *F* = 26.727, *df* = 2, 122, *p* < 0.0001). It also discovered an interaction species*color (GLMM, *F* = 10.659, *df* = 6, *p* < 0.0001). No significant differences between species, sexes and no effect of an interaction species*sex were found.

Goffins interacted significantly more with yellow objects than with red (Wilcoxon test, *Z* = 2.97, *p* = 0.003). Kea preferred yellow to red (Wilcoxon test, *Z* = 2.441, *p* = 0.016) and blue objects (Wilcoxon test, *Z* = 2.201, *p* = 0.02), significantly above chance after Bonferroni-Holms correction). No other significant colour preferences were found (Wilcoxon test lowest *p* value above respective Bonferroni threshold = 0.0167).

The two GPs that did interact with the objects picked stick shaped objects more than other shapes (38.32% Mausi, 37.7% MP, see Figure S1). One of the two BPCs (Madame D.) picked balls more than other objects (47%).

The GLMM for shape preferences for the remaining species (Goffins, kea, YBAs, BBAs) detected differences in the mean percentage of picking a shape between the different shapes (GLMM, *F* = 13.157, *df* = 3, 164, *p* < 0.0001) and an effect of an interaction species*shape (GLMM, *F* = 4.299, 9, 164, *p* < 0.0001). No significant differences between the sexes or an effect of an interaction species*sex were found.

Within species, Goffins chose sticks significantly more than cubes (Wilcoxon test, *Z* =2 .988, *p* = 0.003). Furthermore, kea interacted with balls more than with rings (Wilcoxon test, *Z* = 3.04, *p* = 0.002) and YBAs chose sticks, balls and cubes more than rings (Wilcoxon tests, *Z*_sticks_ = 2.845, *p* = 0.004; *Z*_balls_ = 2.665, *p* = 0.008; *Z*_cubes_ = 2.825, *p* = 0.005). The juvenile kea Paul, which lacked pre-experience with inserting compact objects into tubes in a foraging context also had a preference for balls: 46.1% of all objects he interacted with were ball-shaped. No other differences in shape preferences within the species were found (Wilcoxon tests, *p* values above initial value of respective Bonferroni threshold = 0.008).
